# Are older patients’ cardiac rehabilitation needs being met?

**DOI:** 10.1111/j.1365-2702.2009.02798.x

**Published:** 2009-07

**Authors:** Elizabeth P Tolmie, Grace M Lindsay, Tim Kelly, Debbie Tolson, Susan Baxter, Philip R Belcher

**Affiliations:** Clinical Research Nurse, Glasgow Royal InfirmaryGlasgow, UK; Reader in Clinical Nursing, Nursing Midwifery and Community Health, Glasgow Caledonian UniversityGlasgow, UK; Head of the Social Work Division, Glasgow Caledonian UniversityCowcaddens Road, Glasgow, UK; Associate Dean, Research and Knowledge Transfer, Nursing, Midwifery & Community Health, Glasgow Caledonian UniversityGlasgow, UK; Health Services Researcher, NHS Quality Improvement ScotlandGlasgow, UK; Department of Cardiac Surgery, University of GlasgowGlasgow, UK

**Keywords:** cardiac rehabilitation, illness representations, nurses, older people

## Abstract

**Aims.** The primary aim of this study was to examine the needs of older people in relation to cardiac rehabilitation and to determine if these were currently being met. A secondary aim was to compare illness representations, quality of life and anxiety and depression in groups with different levels of attendance at a cardiac rehabilitation programme.

**Background.** Coronary heart disease accounted for over seven million cardiovascular deaths globally in 2001. Associated deaths increase with age and are highest in those older than 65. Effective cardiac rehabilitation can assist independent function and maintain health but programme uptake rates are low. We have, therefore, focussed specifically on the older patient to determine reasons for the low uptake.

**Design.** Mixed methods.

**Methods.** A purposive sample of 31 older men and women (≥65 years) completed three questionnaires to determine illness representations, quality of life and anxiety and depression. They then underwent a brief clinical assessment and participated in a face-to-face audio-taped interview.

**Results.** *Quantitative:* Older adults, who did not attend a cardiac rehabilitation programme, had significantly poorer personal control and depression scores (*p* < 0·01) and lower quality of life scores than those who had attended. Few achieved recommended risk factor reduction targets. *Qualitative:* The three main themes identified as reflecting the views and experiences of and attendance at the cardiac rehabilitation programme were: ‘The sensible thing to do’, ‘Assessing the impact’ and ‘Nothing to gain’.

**Conclusions.** Irrespective of level of attendance, cardiac rehabilitation programmes are not meeting the needs of many older people either in terms of risk factor reduction or programme uptake. More appropriate programmes are needed.

**Relevance to clinical practice.** Cardiac rehabilitation nurses are ideally placed to identify the rehabilitation needs of older people. Identifying these from the older person’s perspective could help guide more appropriate intervention strategies.

## Introduction

Cardiovascular disease (CVD) accounted for almost 16·5 million deaths globally in 2001 and is now more prevalent in India and China than in all economically developed countries combined (WHO 2002). Every year, around 20 million people survive a stroke or myocardial infarction (WHO 2003) and it is estimated that by 2050, over one billion people will die from CVD disease (Rogers 2004). Over one-third of all cardiovascular deaths are a result of coronary heart disease (CHD) (Mackay & Mansah 2006).

A study recently conducted in the UK has demonstrated that cardiovascular disease and associated risk factors in older women is higher than previously documented (Lawlor *et al.* 2003). The study which used data from 4236 women aged 60–79 years also indicated that the prevalence of CVD was highest in Scottish women when compared to women living in England or Wales. Within Scotland, incidence rate is 1144 per 100,000 for those age 65–74 years and 2189 per 100,000 for those aged 75 and older (2006 age standardised figures). Associated deaths in those aged 64–75 are around 600 and 280 per 100,000 for men and women respectively. This figure increases in those older than 75 years to around 1850 per 100,000 for men and 1300 per 100,000 for women (ISD 2006). Associated morbidity such as angina and heart failure affects around 35% of those aged between 65–74 years of age and 27% of those over the age of 74 years (BHF 2005).

The World Health Organisation has emphasised the need to focus interventions on those at greatest risk (WHO 2003). As people over 65 have a higher prevalence of heart disease and a greater number of modifiable risk factors than younger adults (Fair 2003), addressing this problem is likely to be a cost effective strategy. Effective rehabilitation services have been shown to reduce cardiac death and improve exercise tolerance, functional capacity and quality of life in older people (Lavie & Milani 2000, Ades *et al.* 2002, Hage *et al.* 2003, Marchionni *et al.* 2003, Jolliffe *et al.* 2005).

In a randomised controlled trial, conducted with 270 adults, 90 of whom were older than 75 years, total physical work capacity (as measured bicycle ergometry) and quality of life (as measured by the Sickness Impact Profile) was improved in all age groups at six and 12 months after cardiac rehabilitation. No improvement was noted in those who had had no cardiac rehabilitation (Marchionni *et al.* 2003). Despite the potential benefit that cardiac rehabilitation has for older people, they constitute one of the most underrepresented groups within the programmes currently available (Spencer *et al.* 2001, NHS 2007). For women and men over 62 years of age, attendance rates as low as 14% and 25% respectively have been reported (Ades *et al.* 1992a,b). There is, therefore, a clear need to increase the uptake of cardiac rehabilitation among this population.

While many studies have investigated cardiac rehabilitation and programme uptake rates, only some have included older people in their study population (Clark *et al.* 2004, Witt *et al.* 2004, Cooper *et al.* 2005). Moreover, few have focussed specifically on the older adults’ perspective. We examined older peoples’ perceptions of cardiac rehabilitation and tried to identify the factors that influenced attendance.

## Aims

To examine the needs of older people for cardiac rehabilitation and determine whether these were currently being met. We also sought to identify whether older adults who attended a formal cardiac rehabilitation programme had different views of their illness and of cardiac rehabilitation than those who did not attend. The study focussed on Phase III of the cardiac rehabilitation programme.

## Study design

We used a two stage mixed-methods descriptive approach. Stage 1 used structured questionnaires to identify illness representations, quality of life and anxiety and depression. Stage 2 comprised a brief clinical assessment to identify presence of risk factors. Face-to-face audio-taped in-depth interviews explored experiences of and beliefs about myocardial infarction and cardiac rehabilitation. All data were collected by experienced health care researchers.

## Ethical approval

Ethical Approval for the study was obtained from the appropriate NHS Research Ethics Committee.

## Definition of attendance at cardiac rehabilitation

At the time of the study, none of the participating hospitals were using the Heart Manual© (Lewin 2001). Each participating hospital offered a four-phase cardiac rehabilitation programme. Phase III comprised 24 one hour, twice weekly exercise sessions, delivered within a hospital out-patient facility. Educational sessions and counselling were also available. As the definition of programme attendance differed across the participating sites it was defined as follows:

- Full attendance (24 sessions attended);
- Partial Attendance (at least one session attended);
- Non-attendance (no sessions attended).

## Methods

### Study population

The target population was adults aged 65 years or over, who were able to give informed and competent consent after suffering a myocardial infarction in the previous six months. A purposive sample of participants who met these criteria was selected from the hospital records at the participating sites.

### Study recruitment

Sixty patients with different levels of attendance (full attendance, partial attendance, non-attendance) were identified. Participants were invited to attend only after their General Practitioner had confirmed that they had no health problems that would prevent them from doing so. Invitation was by letter and follow-up telephone call. Of the 60 patients invited, 31 (52% rounded figure) accepted. All completed a written consent form indicating their agreement to take part in the study. No further contact was made with those who declined.

### Data collection

#### Quantitative

Demographic data were collected via case record review. We used the Revised Illness Perceptions Questionnaire (IPQ-R) (Moss-Morris *et al.* 2002) to assess illness representations, the MacNew Heart Disease Health-Related Quality of Life questionnaire (Hofer *et al.* 2004) to assess quality of life and the Hospital Anxiety and Depression Scale (HAD) (Snaith & Zigmund 1994) to assess anxiety and depression. These were mailed to participants who were asked to complete them before the interview and returned by post or collected at the interview.

A brief clinical assessment was conducted after the interview to identify the presence of coronary heart disease risk factors [smoking, elevated plasma cholesterol, elevated blood pressure, central obesity and body mass index (BMI kg/m^2^), presence of diabetes]. The coronary heart disease risk reduction target levels, used nationally at the time of the study (SIGN 41 2000, BCS 2005), determined the extent to which participants were achieving target level.

#### Qualitative

**Figure 1:**
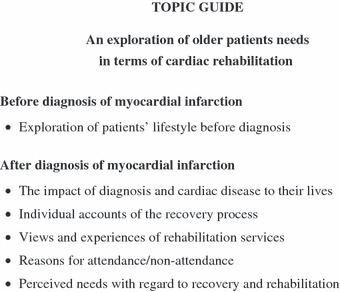
Topic guide.

Most interviews were conducted at the participant’s home (*n* = 29). One was conducted in a designated room within the hospital and another in the university as requested by the participants. Interviews lasted between 30–60 minutes and were audio-taped. We used a brief Topic Guide (Fig. 1) to ensure that key areas of the enquiry were explored; otherwise participants were free to focus on the issues most important to them.

#### Data analysis

***Quantitative:*** Numerical data were analysed using StatsDirect (Altrincham, Cheshire, UK) statistical software. Where data distributions did not follow the normal distribution, data were analysed by ordinal methods and adjusted for ties (Kruskal–Wallis 1-Way anova and Mann–Whitney tests). Results are shown as median and interquartile range (IQR). Significance level was set at *p* ≤ 0·05.

***Qualitative:*** Qualitative data were analysed using Framework Analysis (Ritchie & Spencer 1994). This is a systematic, iterative and inductive process (Huberman & Miles 2002) which facilitates comparison between cases and themes while maintaining the integrity of individual responses (Green & Thorogood 2004). Initially, all audio-tapes were listened to, transcribed verbatim and checked for accuracy. Key issues and themes were identified and data annotated to identify common and divergent themes within and across groups. Data were imported into the qualitative software programme nudist 5® (QSR International, Doncaster, Victoria, Australia) to allow data to be retrieved easily and to aid analysis.

## Results

### Quantitative data analysis

#### Demographics

Sixteen men and 15 women, with an age range of 66–90 years (mean 74·5, SD 6·2) participated in the study. One resided in a care home and 20 (13 men) were living with a partner or family member. Sixteen had participated in a Phase III cardiac rehabilitation programme and 15 did not attend. Of these, four (two women and two men) had not been invited to attend. The ‘not invited’ group were similar to those in the invited groups in respect of the demographic variables. It was not documented in the hospital record that they had not been invited to attend and furthermore they did not know why they had not been given the opportunity. This group was excluded from further analysis.

Of the 27 respondents invited to attend a cardiac rehabilitation programme, nine had completed the programme, seven attended at least one session but did not complete the programme and 11 did not attend any of the programme. We compared these groups on sex, age, deprivation category (McLoone 1994), living alone or with others and programme provider. As shown in Table 1, there were no significant differences between the three invited groups in regard to any of these variables.

**Table 1:** Demographic characteristics of the study participants at each of the hospital sites

	Non-attenders (*n* = 11)	*p*	Partial attenders (*n* = 7)	*p*	Full attenders (*n* = 9)	Non-attenders vs. full attenders (*p*)
Sex (M:F)	6:5	0·54	2:5	0·31	6:3	0·80
Age						
Mean ± SD	75·4 ± 5·1	0·27	72 ± 4·3	0·99	74·4 ± 7·4	0·75
60–69	1	0·96	1	0·93	2	0·98
70–79	8		5		4	
≥80	2		1		3	
Home circumstances						
Alone	4	0·67	2	0·95	2	0·43
With partner/family	7		5		7	
Residential care home	0		0		0	
Deprivation category Median [IQR]	4 [2–6]	0·56	2[2–6]	0·49	5 [3–6]	0·97
1–2	3	0·90	4	0·67	2	0·94
3–5	3		1		3	
6–7	4		2		4	
Unknown	1					
Programme provider: hospital sites						
1	3	0·83	1	0·99	1	0·79
2	3		2		3	
3	5		4		5	

#### Clinical assessment – risk factors

We compared each group in respect of the main CHD risk factors and the extent to which they exceeded guideline recommendations. We examined each group independently as well as in combination. Results are presented in Table 2. As shown, when individual groups were compared, no statistically significant differences were detected in any of the indices measured (*p* > 0·05). When groups were combined and contrasted by level of programme attendance (none, partial, complete) significant between group differences were detected in systolic blood pressure and male girth.

**Table 2:** Level of cardiac rehabilitation attendance in relation to coronary heart disease risk factors

Level of programme attendance	Non-attenders (*n* = 11)	*p*	Partial attenders (*n* = 7)	*p*	Complete attenders (*n* = 9)	Non attenders vs. complete attenders *p*
*Risk Factor*						
Total cholesterol (mmol) Median [IQR]	3·3 [3·3–3·6]	0·98	3·6 [3·0–3·6]	0·63	3·4 [2·8–4·1]	0·90
*n* > 5 mmol	0		1		0	
Systolic blood pressure (mmHg) Median [IQR]	121 [120–130]	0·10	142 [130–162]	0·33	130 [128–148]	0·67
*n* > 140 mmHg M:F	1:0		2:2		2:2	
Diastolic blood pressure (mmHg) Median [IQR**]**	67 [58–80]	0·99	74 [58–80]	0·99	76 [69–79]	0·99
*n* > 90 mmHg M:F	0:0		0:0		0:0	
Blood pressure Mean, ±SD	88*·*2 ± 11*·*4	0·28	96·4 ± 10	0·95	93*·*3 ± 8·2	0·53
Pulse pressure Mean, ±SD	57·6 ± 20·7	0·34	73·1 ± 17·1	0·31	58·7 ± 11·7	0·96
*n* > 60 mmHg	3:2	0·09	2:2	0·07	1:2	0·08
Body Mass Index Mean, ±SD	26·4 [3·8]	0·53	21·7 [1·4]	0·26	33·4 [10·0]	0·47
	29·1 [4·8]	0·99	29·6 [2·8]	0·53	27·2 [2·8]	0·99
*n* > 25 M:F	5:4		2:5		5:3	
Waist circumference (cms) Mean, ±SD	99 ± 9	0·54	88 ± 2	0·12	107 ± 4	0·42
	98 ± 13	0·93	93 ± 13	0·99	89 ± 16	0·72
*n* > 102 (M), > 88 (F) M:F	3:4		0:3		5:0	
Waist Hip Ratio Mean, ±SD	0·95 ± 0·06	0·54	0·89 ± 0·03	0·64	0·96 ± 0·08	0·99
	0·81 ± 0·05	0·99	0·83 ± 0·07	0·99	0·83 ± 0·08	0·98
*n* > 0·95 (M), 0·80 (F) M:F	3:3		0:3		3:2	

The non-attender group had significantly (*p* = 0·02) lower systolic blood pressure (121 [120–130] mmHg) compared to that of the combined partial and full attender group (140 [129–154] mmHg). In addition, men in the full attender group were found to have a larger mean girth (107 SD 4) cm than that of the combined non-attender and partial attender groups (96 SD 9) cm (*p* = 0·04). No other differences were detected in other combined and contrasted groups.

#### Numbers exceeding recommended target levels for risk reduction

All but one respondent achieved the recommended target level for total cholesterol and all achieved the recommended target for diastolic blood pressure. Self-reported alcohol consumption was low with only one (male) exceeding the recommended limit. Other risk factors were less well controlled. Six of seven smokers had continued to smoke. Three were non-attenders (Table 2).

There was no significant association between level of attendance at a cardiac rehabilitation programme and achievement of recommended target for systolic blood pressure (*p* = 0·20), pulse pressure (*p* = 0·47), body mass index (*p* = 0·30), waist circumference (*p* = 0·26), or waist–hip ratio (*p* = 0·88).

#### Co-existing health problems

A wide range of co-existing health problems were reported with non-attenders reporting more problems (Median = 2 [1–4]) than both partial (Median: 1 [0–3]) and full attenders (Median: 1 [0–2]). However, the difference was not statistically significant (*H* = 0·35 adjusted for ties).

Co-existing health problems were categorised by type and the presence of the main concomitant symptoms (breathlessness/angina, vascular problems mobility problems) compared across attendance groups. No statistically significant differences in breathlessness (χ^2^ = 1·18, df = 2, *p* = 0·64), vascular (χ^2^ = 0·97, df = 2, *p* = 0·61) or mobility problems (χ^2^ = 0·83, df = 2, *p* = 0·68) were found.

#### Anxiety and depression

Overall, 13 (43%) participants had borderline or above anxiety (HAD) scores (score > 7) (Median 5·5, [3–11]). The proportion whose anxiety scores were indicative of clinical caseness (χ^2^ = 0·80, df = 2, *p* = 0·78, (Fisher’s exact test) did not differ significantly when compared by attendance. However when groups were split by sex and compared, women were found to have poorer scores (Median 9 [5–12] than that of men (Median 3 [1·5–6·5]). The difference was statistically significant (CI −8 to −1) (*p* = 0·019).

Seven (23%) participants had borderline or above depression scores (score > 7) (Median 5, [2–7]). The scores of non attenders were poorer than those of attenders (*p* < 0·01), although the proportion with scores indicative of clinical caseness did not differ significantly (χ^2^ = 4·8, df = 2, *p* = 0·17) (Fisher’s Exact test). Depression scores of men (median 4·5 [2–7]) were not significantly different to that of women (median 6 [3–7]) (*p* = 0·71, 95% CI −3 to 2).

#### Quality of life

Quality of life (QoL) was measured using the four dimensions of the MacNew heart disease health related QoL questionnaire which comprises four dimensions (physical, social, emotional, global). There were no statistically significant differences in any of the dimensions when individual groups were compared or when groups were combined and contrasted. However, non-attenders had lower scores (all dimensions) than partial attenders and completers (see Table 3).

**Table 3:** Median and inter-quartile [IQR] MacNew scores

MacNew	Non-attenders (*n* = 10)	*p*	Partial attenders (*n* = 7)	*p*	Complete attenders (*n* = 9)	Non-attenders vs. full attenders (*p*)
Emotion	5 [3*·*6–5*·*7]	0·62	5*·*6 [4*·*0–6*·*7]	0·99	5*·*5 [5*·*1–6*·*4]	*a 0·49*
Physical	4*·*85 [3*·*3–5*·*4]	0·71	5*·*3 [2*·*8–6*·*5]	0·96	5*·*9 [5*·*3–6*·*2]	*b 0·13*
Social	4*·*35 [3*·*5–5*·*8]	0·77	5*·*3 [2*·*5–6*·*8]	0·77	6*·*1 [5*·*6–6*·*3]	*c 0·12*
Global	4*·*45 [3*·*7–5*·*7]	0·68	5*·*4 [3*·*1–6*·*6]	0·92	5*·*8 [5*·*5–6*·*0]	*d 0·91*

0 = poorer QoL, 7 = better QoL.

#### Illness representations

The individual components of the IPQ-R (symptoms, acute/chronic or cyclical timeline, personal and treatment control, consequences and emotional representations) were used to provide a quantitative measure of illness representations. No statistically significant differences were detected in any component of the IPQ-R when individual groups were compared. However when groups were combined and contrasted as before, non attenders were found to have lower *personal control* scores than that of the combined partial and full attender group (median 21 [8–24] vs. median 24 [23–25·5] respectively). This difference was statistically significant (*p* = 0·02) (95% CI −6 to 0).

Women had stronger treatment control scores than that of men (median 19·5 [18·5–20] vs. median 18 [15–18]). This difference was also statistically significant (p = 0·04 adjusted for ties) (95% CI −5 to 0). No other statistically significant differences were detected in the combined and contrasted groups or between men and women.

### Qualitative data analysis

The three main themes identified as relating to programme attendance were: ‘The sensible thing to do’, ‘Assessing the impact’, and ‘Nothing to gain’. These were ratified by the second author. Findings are reported in the following section.

#### The sensible thing to do

Participants who attended all or some of the sessions within the cardiac rehabilitation programme were, in general, happy with what was provided. Classes had, however, provided little time or opportunity for social interaction and, as illustrated by the comment, ‘the girl [had] turned on the music and left us to it [because] she was probably going to a keep fit class or something’, they may have provided little incentive for continued attendance:

R: 027: I felt well it was part and parcel of your rehabilitation and I thought it silly not to do it …. I felt this would get me back on my feet again. I knew I mean (the doctors) said no badminton, no bowls no nothing until we tell you that you can start doing it again and I was just wanting something to help me get back on my feet again.

There was a belief among attenders that the programme was ‘more suitable for those who don’t do any [exercise]’ but that adhering to treatment recommendations was ‘the sensible thing to do’. Most reported that having ‘always been active’ they had been anxious to ‘get back on [their] feet again’. Completing the cardiac rehabilitation programme was thus considered to be an essential part of their recovery.

#### Assessing the impact

Participants who had started, but not persisted, with the programme constituted two groups; those who attended the first session only and those who attended several sessions before discontinuing. Those in the former group considered the exercise regime to be too strenuous or to be outwith their capabilities. The effort exerted in conducting day to day activities was as much as they felt capable of managing. As illustrated below, some had experienced severe pain or exhaustion during or after the exercise session:

R: 004:  I used to say you’ll need to [slow] the music down, it’s going too quick for me … to tell you the truth I really enjoyed it, but when I came out of there, I couldn’t walk… I was crying…with the pain. Oh you’ve no idea the pain. … I’m too frightened to try again to tell you the truth…

For some, low-level exercises programmes provided an appropriate alternative. Others believed that the activity associated with such programmes would not provide any more benefit than what would be gained at home while performing routine tasks or everyday activities.

Some of those who attended the programme for several sessions before discontinuing, did so only until they reached a point where they felt ‘back to normal’:

R: 021: I’m more or less doing what I did before. I get tired more quickly but that’s really about the only thing I notice… what they did in the exercise [class] I mean, I think I do more or less exercise em …myself with what I do …. So I didn’t really find that terribly helpful.

Once further health improvement was not evident to them, or when they were no longer challenged by the programme, they discontinued.

#### Nothing to gain

The reasons cited by those who had chosen not to attend the programme were more diverse than those of ‘partial attenders’. At times comments from a family member who believed they weren’t ‘at that stage’ and ‘wouldn’t gain anything’, a consultant who had made them feel ‘worthless’, or a member of the cardiac rehabilitation team who told them they didn’t ‘think there was much [they] could do for them’, had acted as a disincentive. For others, it was physically restricting or socially embarrassing problems such as arthritis or incontinence, or the desire to reduce the time already being spent attending clinics or hospital, that had negatively influenced their decision regarding attendance.

Many held negative beliefs about the ageing process and the extent to which their health and quality of life could be improved. There was a view that, aside from medical intervention, little could be done to prevent the progression of heart disease. Thus, surgical, radiological and pharmacological interventions were perceived as more effective than lifestyle change. As illustrated below, these were viewed as all that was required to permit life to continue as before:

R:00: I’ll be seventy-seven this year. And, old age doesn’t come itself… [I] don’t think I’d be a great deal better… I like being independent. But …see [I] have no family ye know. And eh…[I’m] not really keen on staying too long …I’d rather, eh, live a natural life than to sort of linger on and become a burden to people you know. And eh… when it’s done its done, you know…

R:009 … And I took them (the tablets) and everything’s working okay. I didn’t need anybody really. I’m not maligning the services that they give, but they couldn’t give me anything. The tablets did me fine….I’ve only got the one life and I… intend to use it as it suits me.

Attending a cardiac rehabilitation programme was, therefore, often viewed as an unnecessary and ineffective intervention.

## Discussion

The mixed-method approach used in this study examined cardiac rehabilitation ‘needs’ in the context of respondents’‘felt needs’, anxiety and depression, quality of life and attainment of recommended target levels for coronary heart disease risk reduction (BCS 2005). We found that, irrespective of cardiac rehabilitation programme attendance, older peoples’ needs were not being fully met in these areas.

### Reducing risk

Plasma cholesterol and diastolic blood pressure were within target levels for all but one respondent. This is an improvement on previously published work which reported the persistence of uncorrected risk factors in a population of cardiac patients (Lindsay *et al.* 2003). However, other modifiable risk factors persisted. We cannot explain why, when groups were combined and contrasted by attendance level (full, partial, none), systolic blood pressure was significantly lower in the non-attending group (*p* = 0·02); or why men who completed the cardiac rehabilitation programme had a larger girth than those who had not completed the programme (*p* = 0·04).

Although the small sample size limits the confidence that can be placed in these results, they do merit further investigation. Given that almost all respondents exceeded current guidelines for waist circumference and body mass index and all but one smoker continued to smoke, there is clearly room for improvement with regard to support and advice for risk reduction. As morbidity associated with coronary heart disease is rising in those aged over 65 and is expected to continue to rise worldwide as the population ages (WHO 1999); the issue is how do we increase uptake?

## Co-existing health problems

A wide range of co-existing health problems were reported with some respondents commenting that their reluctance to attend cardiac rehabilitation was a consequence of co-existing health problems that physically limited or embarrassed them. Non-attenders reported more problems (Median: 2 [1–4]) than both partial (Median: 1 [0–3]) and full attenders (Median: 1 [0–2]). The difference was not significant (*H* = 0·35 adjusted for ties). As the participants in this study all had vascular disease (by definition) it is not surprising that so many co-morbidities exist. Physical symptoms, lack of energy and painful joints, however, have a significantly negative impact (*p* < 0·01) on the extent to which older people participate in physical activity (Crombie *et al.* 2004). While statistically, the presence of such problems did not appear to influence the degree of attendance, the inability to cope with or accommodate additional health problems, rather than the severity of the problem itself, may prohibit attendance. As we did not objectively measure either the impact of co-existing disease or coping, additional research would be required to determine whether this is so.

The presence of co morbidities does not necessarily exclude older people from participation in a cardiac rehabilitation programme (Oldridge & Stump 2004); the benefits of which should not be underestimated. Patients who have heart disease as well as other co morbidities may be at greater risk of reduced activity and increased dependence (Oldridge & Stump 2004). Appropriate exercise training enables daily activities such as stair climbing, household chores and physical leisure pursuits to be undertaken without angina or dyspnoea (Kavanagh *et al.* 2002). Moreover, several studies have shown that older people who exercise have a reduced risk of falls, deep vein thrombosis, immobility and constipation (Fiatarone *et al.* 1994, Young & Dinan 1994, Campbell *et al.* 1997).

Recent guidelines published by the National Institute of Clinical Effectiveness state that existing problems which restrict physical activity should be addressed before patients are offered cardiac rehabilitation and the exercise component modified to accommodate individual needs (NHS 2007). Addressing problems that limit physical activity may encourage attendance.

## Anxiety and depression

A higher rate of anxiety among women compared with men is a well recognised feature of cardiac disease (Hofer *et al.* 2004) and was supported by our own findings where women had significantly higher anxiety scores than men. In addition, non attenders had poorer depression scores than attenders (*p* < 0·01). While previous studies have shown depression to be a known risk factor for patients with cardiac disease (Mendes de Leon *et al.* 2001, Dobbels *et al.* 2002, Bjerkeset *et al.* 2005) and to be more prevalent in older populations, exercise has been shown to improve depression scores, mood and somatisation (Kavanagh *et al.* 2002).

## Quality of life

A recent review of cardiac rehabilitation studies suggested that comprehensive cardiac rehabilitation programmes after MI did not have any significant effect of on QoL outcomes (NHS 2007). In this study, however, non-attenders had poorer QoL scores on all dimensions (emotional, physical, social, global) (MacNew) than those who had attended. Although not statistically significant, the results may be clinically significant in that non-attenders may be those who are most in need of the support they provide. This may explain, to some extent, why the overall incidence of anxiety and depression we found in both women and men was higher than that published elsewhere (Dixon *et al.* 2000, Cameron & Leventhal 2003). However, depression scores of non attenders were poorer than those of attenders (*p* < 0·01). As previous research on older people who do not attend cardiac rehabilitation is limited, we do not know whether lower mood prohibits programme attendance but it seems likely that it is a contributory factor. Given that anxiety and depression scores may improve with cardiac rehabilitation (Lewin *et al.* 1992, Ades *et al.* 2001, Michie *et al.* 2005), improving uptake may reduce the prevalence of anxiety and/or depression in this population.

Addressing age-related problems known to be linked to depression (such as hearing loss) (Tolson *et al.* 2002, Knussen *et al.* 2004), may also improve both uptake and outcome.

### Illness representations

Illness representations are thought to determine what action a person will take to deal with their health problem and the extent to which they believe outcome can be influenced (Weinman *et al.* 1996, Petrie *et al.* 2002). In this study, personal control beliefs were significantly stronger in those who attended the cardiac rehabilitation programme (*p* = 0·02). It was not possible to determine whether programme attendance strengthened feelings of personal control or whether stronger personal control beliefs promoted attendance. Further research is needed to identify which is the case. However, older people who perceive themselves to have control over their future health have been shown to be more likely to make modifications to their lifestyle than those who feel they have little or no control (Campbell *et al.* 1995, Michie *et al.* 2005). Finding ways to increase feelings of personal control may promote positive health behaviour. Interestingly, women had stronger ‘treatment control’ beliefs than men. This may explain, to some extent, the lower reported programme attendance in women compared to men (Young & Kahana 1993) and merits further investigation. It is possible that women are more inclined to perceive cardiac rehabilitation programmes as irrelevant, or ineffective, in terms of health gain and view medical intervention as the only feasible option.

## Conclusion and recommendations

Our findings support those of Moore (1996) and, more recently, Davidson *et al.* (2003) in that older people desire cardiac rehabilitation programmes that they perceive as being more conducive to their needs. Having more time within the programme to talk and interact with others, a more supportive environment and more choice in what type of activity to invest their time were important. Activity choices were selected in line with personal preference and beliefs about what would best meet personal need. While some of these activities may provide all that is required in terms of rehabilitation, others are likely to be inadequate in terms of improving health and reducing future risk. Offering a choice of activities and promoting activities that can be integrated into daily life may address this issue. Effectively communicating the benefits of cardiac rehabilitation in a way that is meaningful to older people such as the increased independence, ability to perform daily household chores and pursue preferred leisure activities may be a helpful strategy. Incorporating strategies to enhance feelings of personal control and reduce levels of anxiety and depression may also be helpful.

Further investigation would be required to determine if this would be an effective strategy. However, as there was a desire to reduce time spent at hospital or clinics, a re-design of the service which takes account of wider health and social needs may prove beneficial. Programmes situated near transport links may help maintain independence (NHS 2007). Venues located in areas frequented by older people and which provide social space and opportunities for interaction may promote attendance by encouraging people to view the service as social rather than clinical provision. Providing patients with greater opportunity for social interaction (NHS 2007) and facilitating more interaction with staff may also help ensure that those who do enroll in the programmes continue with them.

Cardiac rehabilitation programmes have the potential to improve QoL and help maintain independence. At present, however, they are not meeting the needs of many older people either in terms of risk factor reduction or programme uptake. As the reasons for low uptake among older adults may differ from that of younger adults, understanding the reasons for their decision in respect of attendance may help guide the development of new programmes and, in doing so, increase uptake rates and improve the health of older people. To date, there is no evidence of interventions to improve either uptake or adherence to cardiac rehabilitation in older people (NHS 2007). Further research in this area is required. Programmes which include a social component and which incorporate a psychological element to help older people cope with their diagnosis and the effects of their MI in conjunction with pre-existing health problems may be effective. In addition, investigations should be conducted to identify why, despite recommendations that everyone admitted to hospital with a MI should be offered the opportunity to attend a rehabilitation programme, some older people are not invited to do so.

The findings of this study may be applicable to other chronic diseases such as osteoarthritis and chronic obstructive pulmonary disease that are prevalent in older people and increase their risk of dependency.

## Limitations of the study

The sample size in this study was smaller than anticipated and risk factors for coronary heart disease were measured on only one occasion. Therefore, results from the quantitative component of the study should be treated with caution, although high degrees of statistical difference were seen. Using a larger sample for the quantitative component of the study may have allowed more definitive conclusions to have been made. However, for the qualitative component of the study, the sample was appropriate and, although the study aimed to identify whether older peoples’ needs in terms of cardiac rehabilitation were being met, it is likely the findings are applicable to older people with other chronic health conditions. Moreover, the mixed-method design enabled us to review results from the quantitative component of the study alongside the findings from the qualitative data, thereby providing a more complete and representative picture of older adults’ beliefs about cardiac disease and the factors that impact on their decisions regarding their participation in a cardiac rehabilitation programme. However, due to inadequate power of the study, no firm conclusions can be drawn.

## Relevance to clinical practice

Cardiac rehabilitation nurses are ideally placed to identify the rehabilitation needs of older people. Identifying these from the older person’s perspective could help guide more appropriate intervention strategies.
